# The visual function assessment: from birth to the follow up

**DOI:** 10.1186/1824-7288-41-S1-A34

**Published:** 2015-09-24

**Authors:** Daniela Ricci, Domenico M Romeo, Eugenio Mercuri

**Affiliations:** 1 Pediatric Neurology Unit, Catholic University, Rome, Italy; 2 National Center for Services and Research for the Prevention of Blindness and Rehabilitation of the Visually Impaired - IAPB Italia onlus, Italy

## Background

Preterm infants have a high risk to develop visual deficits due to retinopathy of prematurity (ROP), brain lesionsand prematurity per se [1]. The possibility to assess different aspects of visual function can allow early and specific intervention in an attempt to reduce the risk of difficulties in motor coordination, attention and learning at school age. 

The aim is to identify early signs of visual and motor-perceptual deficit in the first years in order to program a specific intervention before school age.

## Methods

Verypreterm infants born at Gestational Age (GA) <31 weeks, with and without brain lesions and ROP ≤ stage 2, were assessed at 35 and 40 weeks post-menstrual age using a visual assessment specifically designed for neonates; a structured follow up assessment, including fixing, tracking, visual acuity, visual fields and visual attention (using the Fixation Shift test) was used at 3, 5 and 12 months corrected age. Tractography of the optical radiation was performed in some consecutive infants in the neonatal period. Results at all the tests were compared with normative data on term born infants.

## Results

Ocular movements and tracking were more complete in preterm infants at 35 weeks than in full term infants, whereas reaction to a colored target, discrimination of stripes and attention at distance were more mature at term age both in preterm and term born infants. Tractography of the optical radiation showed that at term equivalent age visual assessment was significantly correlated with fractional anisotropy values (*P*<0.001).At 3, 5 and 12 months preterm infants showed similar results to term born infants in all visual aspects but visual attention, with a high percentage of infants failing or refusing the test. Irrespective from the MRI findings, preterm infants with a normal neonatal assessment showed normal visual competences at 12 months corrected age.

## Conclusions

A structured visual assessment can be reliablesince the neonatal age [2]. Some visual aspects are influenced by extrauter ineexperience, others depend on cortical maturation [3] as proved by the level of development of the white matter in the optical radiation [4]. The neonatal assessment has a good correlation with visual development at one year [5]. In low-risk preterms visual attention appears to be already impaired in the first year from birth [6]. 
